# Randomised double-blind crossed-over control trial to determine the therapeutic effect of Nikken® magnetic insoles (Magsteps®) in the treatment of plantar fasciitis

**DOI:** 10.1186/1757-1146-8-S2-P15

**Published:** 2015-09-22

**Authors:** Paul Tinley, Amanda Meneghello, Michelle Hey

**Affiliations:** 1 Charles Sturt University, Albury, NSW 2640, Australia; 2 Leeming Podiatry Centre 56 Farrington Rd Leeming WA 6149, Australia; 3 Charles Sturt University, Wagga Wagga, NSW 2650, Australia

**Keywords:** Magnetic insoles, plantar fasciitis

## Background

The purpose of this study was to determine the therapeutic effect of magnetic insoles in the treatment of plantar fasciitis. This investigation is warranted due to the extensive claims being made in the media regarding the effects of magnets on health and the wide availability of magnetic insoles to the general public.

## Methods

This research was conducted using a randomised double-blind crossover design with ten patient volunteers, using ten pairs of Nikken® magnetic insoles (Magsteps®) Gauss strength 450 and ten pairs of identical sham insoles (non magnetized). Pain and disability were evaluated using the Foot Function Index.

## Results

Magnetic insoles provided no statistically significant therapeutic effect in the treatment of plantar fasciitis, F (2, 16) = 0.14, p = 0.875.

## Conclusions

While statistical analysis did not show any statistical difference, all subjects in the study reported some level of relief of symptoms during the magnetic insole phase of therapy. This was further supported by anecdotal evidence of all subjects wishing to continue using the magnetic insoles after the study had finished.

